# Vision Measurement of Tunnel Structures with Robust Modelling and Deep Learning Algorithms

**DOI:** 10.3390/s20174945

**Published:** 2020-09-01

**Authors:** Xiangyang Xu, Hao Yang

**Affiliations:** 1 School of Rail Transit, Soochow University, Suzhou 215006, China; X.Y.Xu@suda.edu.cn; 2 School of Civil Engineering and Architecture, Jiangsu University of Science and Technology, Zhenjiang 212003, China

**Keywords:** tunnel inspection, 3D modeling, crack detection, camera array, robust modelling

## Abstract

The health monitoring of tunnel structures is vital to the safe operation of railway transportation systems. With the increasing mileage of tunnels, regular inspection and health monitoring are urgently demanded for the tunnel structures, especially for information regarding deformation and damage. However, traditional methods of tunnel inspection are time-consuming, expensive and highly dependent on human subjectivity. In this paper, an automatic tunnel monitoring method is investigated based on image data which is collected through the moving vision measurement unit consisting of camera array. Furthermore, geometric modelling and crack inspection algorithms are proposed where a robust three-dimensional tunnel model is reconstructed utilizing a B-spline method and crack identification is conducted by means of a Mask R-CNN network. The innovation of this investigation is that we combine the robust modelling which could be applied for the deformation analysis and the crack detection where a deep learning method is employed to recognize the tunnel cracks intelligently based on image sensors. In this study, experiments were conducted on a subway tunnel structure several kilometers long, and a robust three-dimensional model is generated and the cracks are identified automatically with the image data. The superiority of this proposal is that the comprehensive information of geometry deformation and crack damage can ensure the reliability and improve the accuracy of health monitoring.

## 1. Introduction

With the fast development of tunnels, cracks and deformations in such structures become an unavoidable problem which will affect their stability and safety and have a serious negative impact on the operation of the train, especially for the high-speed rail. Therefore, it is vital to inspect the status of tunnels structures regularly to avoid accidents resulting from structure failure and to simultaneously extend their lifetime by identifying deterioration at an early stage and performing the maintenance required. However, traditional methods of tunnel inspection are time-consuming, tedious and expensive, and manual inspection is also highly dependent on human subjectivity and exposes inspection personnel to possibly dangerous environments [1].

Nowadays, various regular inspection methods have been introduced to improve tunnel operation safety. Cho et al. [2] employed an octree structure to compress the terrestrial laser scanning data and detect cracks using a combination of image processing algorithms, including K-means clustering, median filtering, binarization, morphological operations and blob removal. Murakami et al. [3] proposed a remote surface measurement system that uses a laser, which focuses on the detection of cracks on tunnel surfaces, to inspect tunnel walls. Afshani et al. [4] investigated the detection rate of defects in concrete lining using an infrared thermography method, where a thermal numerical model is employed to simulate heat transfer mechanics in this site using the field measurement data. Daneshgaran et al. [5] introduces a low-cost technique for road tunnel inspections based on a simple system which does not require complex preliminary work and may also be applied for tunnel health monitoring under the normal traffic flow. Hirata et al. [6] developed a highly sensitive magnetic field detector with a wide frequency range for non-destructive testing with magnetic sensors. The localization in underground spaces and on long linear paths is a challenging task, and the autonomous inspection of tunnels requires advanced capabilities of the robotic vehicle and the computer vision subsystem [7]. Lee et al. [8] developed a shape-sensitive kernel function for a deep learning algorithm and were able to predict the width of cracks by one or two pixels more precisely than the traditional semantic segmentation method. Medina et al. [9] investigated a system for the detection of cracks in concrete tunnel surfaces, based on image sensors where data processing is performed by applying a Gabor filter invariant to rotation, allowing the detection of cracks in any direction.

The geometric inspection of tunnels is gaining increasing attention. Son et al. [10] proposed a method for three-dimensional (3D) modelling for pipelines from laser-scan data which contained three steps: identifying the existence and location, segmenting the surface and reconstructing the geometry. Laefer et al. [11] investigated a means of automatically identifying structural steel members from a terrestrial laser scan point cloud and to generate that geometry in a building information model-compatible format where the proper shape and dimensions of the cross-section are established by employing kernel density estimation. Valero [12] presents a terrestrial laser scanning data processing pipeline aimed at producing semantic 3D models of furnished home and office interiors, which uniquely integrates intelligent technology radio frequency identification that is increasingly used for facilities management. Ullah et al. [13] developed an analytical method which adopted equation- and algorithm-based approaches to generate point cloud data for modelling the 3D object which could be processed by using a commercially available computer-aided design package to create the corresponding virtual models or solid computer-aided design models of the objects. Jeon et al. [14] presented a framework which integrates the results of CNN-based two-dimensional (2D) semantic segmentation applied to dense surface reconstruction for generating semantically segmented triangular meshes of reconstructed 3D indoor scenes using volumetric semantic fusion in the reconstruction process. Yang et al. [15] investigated the free-form surface modelling with B-spline approximation by means of a rank-based method. The reconstruction and interpretation of building interiors based on point clouds and images was investigated in which the development of automatic approaches for reconstructing a 3D model from imagery and/or point clouds can make the process easier, faster and cheaper [16]. Various methods have recently been investigated regarding the deformation analysis of tunnel structures [17,18,19,20,21,22,23]. Wiśniewski et al. [24] proposed a total least squares collocation for determining the vertical deformations treated as random fields. Ziolkowski et al. [25] performed an advanced object shape analysis of a composite foot-bridge that is subject to spatial deformations during the proof loading process. Barazzetti et al. [26] presents the development and implementation of three image-based methods used to detect and measure the displacements of a vast number of points in the case of laboratory testing on construction materials. However, investigation on intelligent identification of tunnel cracks combined with deformation is relatively scarce.

In this paper, a new method is proposed which combines freeform surface modelling based on the maximum likelihood function and crack analysis based on CNN for the inspection of tunnel structures, which is a challenging process due to, for example, the noisy data and data gap. The paper is organized as follows: section one gives an introduction to the background and literature; section two describes the vision-based tunnel measurement system and the image data collected; section three is the data analysis, including 3D geometric modelling, where robust approximation with B-splines is introduced, and crack inspection, where the Mask R-CNN algorithm is employed; and the fourth and fifth sections are the discussion and conclusion, respectively.

## 2. Measurement

A smart vision measurement system, which consists of a digital camera array, LED illumination lamps and a moving vehicle, is applied to collect the image data of the tunnel structure. As the data acquisition unit, each camera captures a segmentation of the tunnel section where the resolution of camera is 0.1 mm at the measurement distance of about 2.5–3 m. Ten digital cameras are arranged in a circle array to cover the whole tunnel cross-section to reconstruct the whole structure. The LEDs are adopted to illuminate the surface of the tunnel to ensure the quality of the image data, considering the visibility issue inside the tunnel, especially for long distance tunnels. The trolley equipped with the cameras and the LEDs is arranged to travel at a maximum speed of 5 km per hour along the tunnel, while images are collected simultaneously and the time interval of the shootings is pre-calibrated before the measurement.

The vehicle’s speed of measurement greatly determines the data quality, therefore, the collected images have a lower quality, such as being blurry due to camera shock and vibration, for a high vehicle speed. However, this study concerns mainly the methods and algorithms for the image data processing; the issues of the measurement system will be focused on in the future. In this measurement, the images collected have a good quality and the resolution is better than 1 mm with the speed around three km per hour, which is enough for the geometric modelling and crack detection.

**Figure 1:**
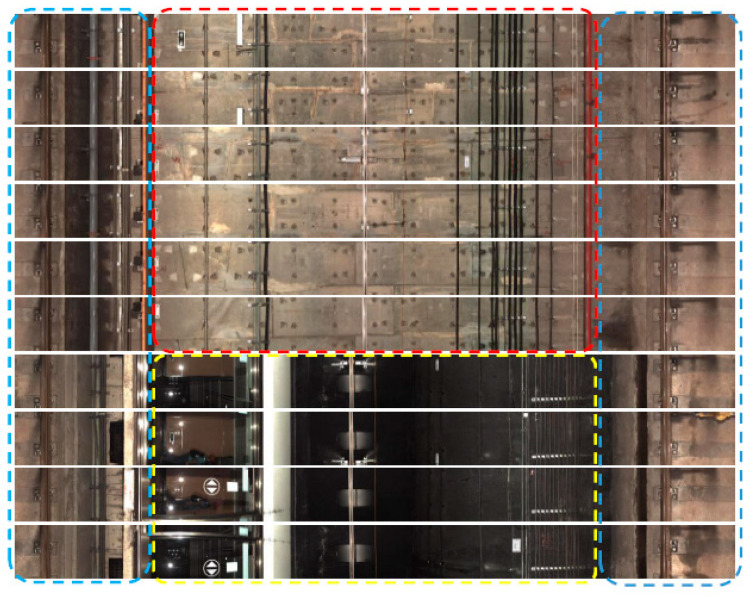
The stitched images of tunnel sections.

In this investigation ten images, which were collected at a certain shooting moment recording the condition of a cross-section circumference of the tunnel, are stitched firstly as a strip for an efficient image processing. An unfolded image is then obtained for the annular region, where some of the unfolded images are presented in Figure 1, in which the middle area marked in red corresponds to the tunnel wall, the two ends marked in blue are the rail area and the area marked in yellow is the platform area.

## 3. Data Analysis

In this section, the analysis of the tunnel measurement data consists of 2D and 3D methods. The 2D analysis is performed with the raw images using computer-vision methods and the 3D exploration is conducted by means of photogrammetry methods. As 2D mapping of the tunnel surface, image becomes a specific way to identify the tunnel cracks, because that optical property, i.e., pixel-wise gray values, of cracks and non-cracks are different, where the gray values of image pixels are treated as input for learning functions, and the output is the decision of whether the pixel represents cracks or not. Moreover, 3D information can be generated through image matching and spatial geometric positioning, thereby the geometric model and deformation of tunnel structures could be obtained.

### 3.1. Robust 3D Modelling

In this study, both 3D and 2D tunnel information are extracted based on the images collected, where images fulfil the overlay condition and can be imported to commercial software to generate 3D point cloud data based on the photogrammetric principle. In order to gain higher accuracy of 3D point cloud data, the image data collected requires adequate information for matching, which depends on the data characteristics and the coincidence rate that is proportional to the moving speed in the directions along the tunnel axis. It is noteworthy that the 3D point cloud is obtained independently from the LIDAR measurements, which reduce the measurement cost, and the accuracy of the 3D point cloud generated can reach mm level locally. Furthermore, the accuracy of the point cloud generated can be improved in a few ways, for example, integrating a LIDAR scanner or installing some targets in the tunnel. The point cloud data is massive point data where the 3D coordinates of each point is known, thereafter, 3D modelling is carried out based on the point cloud data generated by means of the robust B-splines approximation method.

**Figure 2:**
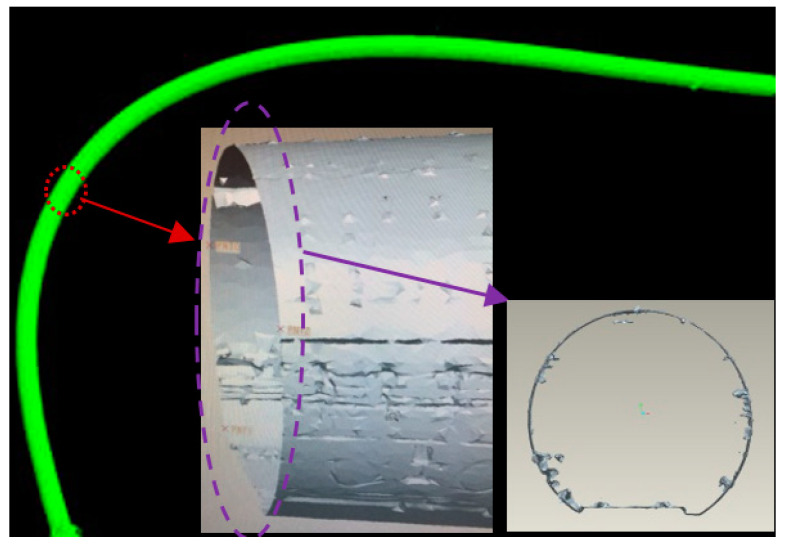
Disturbing objects observed from the 3D model generated by the mesh method.

As we know, the point cloud data are seen by their nature as a huge number of scattered, noisy and unevenly distributed points, while the geometric topography is unrevealed. Therefore, the 3D modelling of the point cloud data is necessary to investigate the geometry and deformation of the tunnel structures. A traditional method used in 3D model reconstruction is the mesh method, which is usually applied in, for example, mine surveys and city planning. The 3D mode using the mesh method is shown in Figure 2, which is obtained by means of commercial software, where the green part is the overall 3D tunnel model and the grey part shows the details of representative tunnel sections. It should be noted that, although the commercial software is capable of generating point cloud and 3D models, the latter are not efficient and accurate enough to show the status of the tunnel. According to Figure 2, it can be observed that many disturbing points exist in the tunnel wall and introduce distortion to the tunnel’s geometric feature model. The disturbing objects, such as electricity lines and rails, do not represent tunnel features themselves. In addition, this 3D model is a complex composition of hundreds of mesh parts, which is computationally expensive for the 3D model operations. Considering this model is not enough for an accurate and refined analysis of the tunnel’s geometric status, therefore, a robust and refined tunnel model is needed for the geometric analysis of tunnel structures.

In this section, B-spline surface approximation is employed to construct a 3D tunnel model in this paper, which is a parametrization representation with a superior flexibility of local approximation compared to other surface forms, such as planes and polynomial surfaces. The goal of the robust B-spline modelling is to construct an automatic and high-accuracy geometric model that is resistant to disturbances and noisy points and only keeps the tunnel features, which is feasible for the deformation analysis of the tunnels. The input for the robust B-spline model is known observations, which is the point cloud data containing the disturbing points, which lead towards a mathematical approximation procedure. Thereafter, the aim is to solve the unknown parameters of the B-spline surface to achieve the best approximation of the estimated B-spline points regarding the geometric features of the tunnel.

The B-spline surface is constructed by a net of B-spline curves. A B-spline curve is constructed by the linear combination of B-spline control points x*_i_* (*i* = 0, …, *n*) and *p*th-degree basis function $N_{i,p}\left(\bar{u}\right)$, which is defined on a location parameter $\bar{u}$, as shown in Equation (1), where $C\left(\bar{u}\right)$ are the B-spline curve points. We use the Cox-de Boor recursive formula to define the basis function $N_{i,p}\left(\bar{u}\right)$, as shown in Equations (2)–(4), where the real numbers $u_{i}$ are a nondecreasing sequence of knots composing knot vector **U**, *m* + 1 is the number of knots, and $\bar{u}$ is the location parametrization of a data point which reflects the position of this data point relative to the sequence of all data points. The parametrization can be determined with a standard chord length method. There are a number of references for detailed explanations of the B-spline basis function, such as [27].

(1) $$C\left(\bar{u}\right)=\sum_{i=0}^{n}N_{i,p}\left(\bar{u}\right)x_{i}$$

(2) $$N_{i,0}\left(\bar{u}\right)=\{\begin{array}{l} 1\;\mathrm{if}\;u_{i}\leq \bar{u}\leq u_{i+1}\\ 0\;\mathrm{otherwise} \end{array}$$

(3) $$N_{i,p}\left(\bar{u}\right)=\frac{\bar{u}-u_{i}}{u_{i+p}-u_{i}}N_{i,p-1}\left(\bar{u}\right)+\frac{u_{i+p+1}-\bar{u}}{u_{i+p+1}-u_{i+1}}N_{i+1,p-1}\left(\bar{u}\right)$$

where

(4) $$U=\left|u_{0},\ldots ,u_{m}\right|\;\mathrm{with}\;u_{i}\leq u_{i+1},i\epsilon \left\{0,\ldots ,m-1\right\}.$$

The B-spline surface is a tensor product as Equation (5), where $S\left(\bar{u},\bar{v}\right)$ represents the B-spline surface, which has two directions of *u* and *v*. The $\bar{u}$, *i*, *p*, *n* in Equation (5) correspond to the location parameter, control point index, degree of Basis function and control point number parameter in the direction of *u*, while $\bar{v}$, *j*, *q*, *m* are those related to the direction *v*.

(5) $$S\left(\bar{u},\bar{v}\right)=\sum_{i=0}^{n}\sum_{j=0}^{m}N_{i,p}\left(\bar{u}\right)N_{j,q}\left(\bar{v}\right)x_{i,j}$$

A linear Gauss–Markov model is combined in order to solve the unknown (*n* + 1) × (*m* + 1) control points x*_i,j_*, which is shown in Equation (6). Here, l is the uncorrelated observations which equal the point cloud data, the unknown **x** is the B-spline control point matrix, **e** is the residuals of the observation, and the design matrix **A** is shaped by the B-spline basis functions as in Equation (7), where *s* and *t* are the total number of points in directions *u* and *v* separately. According to Gauss–Markov model, the estimation of **x** is obtained as Equation (8), where $\hat{x}$ is the estimated control points, and *x_i_*, *y_i_*, *z_i_* are the *x*, *y* and *z* coordinates of these control points.

(6) l=Ax+e

(7) $$A=\left[\begin{array}{ccc} N_{0,p}\left({\bar{u}}_{1}\right)\cdot N_{0,q}\left({\bar{v}}_{1}\right) & \cdots & N_{n,p}\left({\bar{u}}_{1}\right)\cdot N_{m,q}\left({\bar{v}}_{1}\right)\\ \vdots & & \vdots \\ N_{0,p}\left({\bar{u}}_{s}\right)\cdot N_{0,q}\left({\bar{v}}_{t}\right) & \cdots & N_{n,p}\left({\bar{u}}_{s}\right)\cdot N_{m,q}\left({\bar{v}}_{t}\right) \end{array}\right]$$

(8) $$\hat{x}=\left[\begin{array}{ccc} x_{o} & y_{o} & z_{o}\\ \vdots & \vdots & \vdots \\ x_{nm} & y_{nm} & z_{nm} \end{array}\right]={\left(A^{T}A\right)}^{-1}A^{T}l$$

In order to achieve the best B-spline approximation, the control points of B-spline surface can be estimated through three steps. Firstly, the parametrizations acquiring $\bar{u}\;\mathrm{and}\;\bar{v}$ are carried out with the data of s rows and t columns; secondly, knot vectors **U** and **V** are determined in the directions of *u* and *v* through Equation (4); thirdly, the design matrix **A** is computed according to Equation (7) and the control points are estimated with Equation (8).

Because the point cloud data are contaminated with disturbing points, an iteratively reweighted least squares method (IRLS) is employed for a robust estimation, where the estimated B-spline surface best approaches the tunnel geometry. This is achieved by that the observation point with a large residual will be down-weighted with the diagonal weight matrix $P_{ll}$. The estimated $\hat{x}$ considering weight matrix is shown in Equations (9) and (10), where *p_i_* (*i* = 1, …, *n*) is the n diagonal elements of weight matrix.

(9) $$\hat{x}={\left(A^{T}P_{ll}A\right)}^{-1}A^{T}P_{ll}l$$

where

(10) $$P_{ll}=\mathrm{diag}(p_{1},\ldots ,p_{n})$$

Adaptive IRLS method is necessary to achieve the automotive adjustment of weights, where tuning constants are adjusted according to characteristics of the actual data instead of specified in advance of the computation. The variance-inflation model based on the scaled *t*-distribution is used to obtain adaptiveness of the IRLS method [28], with the assumption that the measurement errors of the l follow an independent, identical and centered *t*-distribution $t_{\nu }\left(0,\sigma^{2}\right)$. In order to solve the unknown parameters, a likelihood function of the *t*-distribution is set up with the probability density function of the scaled *t*-distribution which depends on the unknown parameter **x**, degree of freedom ν and the scale factor $\sigma^{2}$. The maximum likelihood estimation of the unknown parameters is conducted and Equations (11)–(14) give the adaptive estimation of **x**, ν and $\sigma^{2}$ with an iterative computation [28], where *k* denotes the *k*th iteration step.

(11) $$p_{i}^{\left(k\right)}=\frac{\nu^{\left(k\right)}}{\nu^{\left(k\right)}+{\left(\frac{l_{i}-A_{i}x^{\left(k\right)}}{\sigma^{\left(k\right)}}\right)}^{2}}.$$

(12) $$x^{\left(k+1\right)}={\left(A^{T}P_{ll}^{\left(k\right)}A\right)}^{-1}A^{T}P_{ll}^{\left(k\right)}l.$$

(13) $${\left(\sigma^{2}\right)}^{\left(k+1\right)}=\frac{1}{n}{\left(e^{\left(k+1\right)}\right)}^{T}P_{ll}^{\left(k\right)}e^{\left(k+1\right)}.$$

(14) $$\begin{array}{l} 0=\log\nu^{\left(k+1\right)}+1-\psi \left(\frac{\nu^{\left(k+1\right)}}{2}\right)+\psi \left(\frac{\nu^{\left(k+1\right)}+1}{2}\right)-\log\left(\nu^{\left(k+1\right)}+1\right)\\ \;+\frac{1}{n}\sum_{i=1}^{n}\left(\log\left[\frac{\nu^{\left(k+1\right)}}{\nu^{\left(k+1\right)}+{\left(\frac{l_{i}-A_{i}x^{\left(k+1\right)}}{\sigma^{\left(k+1\right)}}\right)}^{2}}\right]-\frac{\nu^{\left(k+1\right)}}{\nu^{\left(k+1\right)}+{\left(\frac{l_{i}-A_{i}x^{\left(k+1\right)}}{\sigma^{\left(k+1\right)}}\right)}^{2}}\right) \end{array}$$

**Figure 3:**
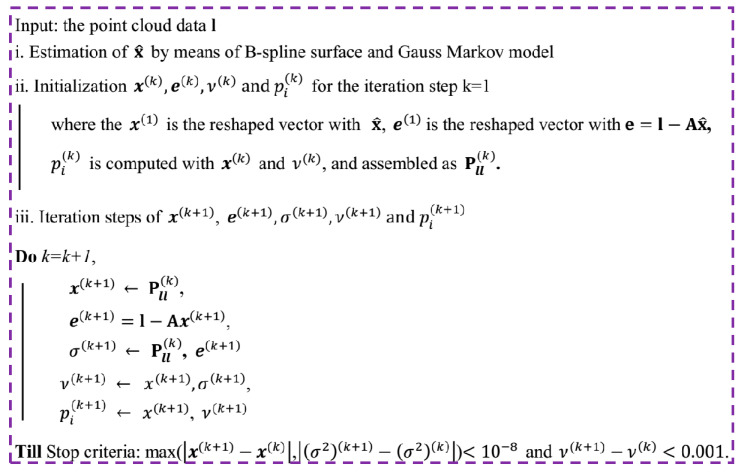
The IRLS algorithm for robust B-spline modelling.

The computational procedures of the robust B-spline modelling are shown in Figure 3, where the IRLS algorithm for robust B-spline modelling is adopted.

In Figure 3, a stop criteria is the maximum of the absolute differences $x^{\left(k+1\right)}-x^{\left(k\right)}$ and ${\left(\sigma^{2}\right)}^{\left(k+1\right)}-{\left(\sigma^{2}\right)}^{\left(k\right)}$ are less than 10^−8^ and the difference $\nu^{\left(k+1\right)}-\nu^{\left(k\right)}$ is less than 10^−3^. The computation of the new degree of freedom in the (*k* + 1)th step $\nu^{\left(k+1\right)}$ is carried out through the solution of Equation (14), where ψ is the digamma function.

The robust 3D parametric reconstruction of tunnel structures is achieved in which the unknown control point coordinates x are estimated with the adaptive IRLS method. Because the robust estimation is based on the weight optimization of the Gauss–Markov model, main parameters of the number of control points and degree of B-spline basis function were determined in the step of Gauss–Markov estimation. In this study, the determination of the number of control points assures 2 m distance between two neighboring control points, and the degree of B-spine function is 2, which are tested to be fit the curvature of the tunnels and the less computational consumption.

### 3.2. Crack Analysis

**Figure 4:**
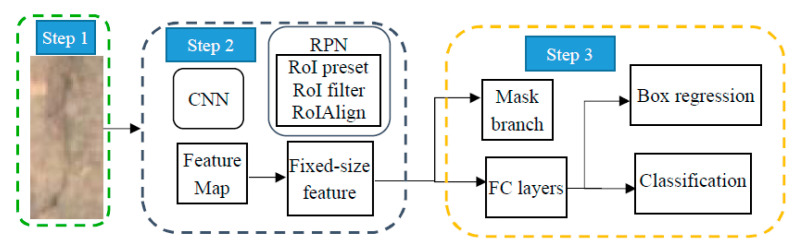
The procedure of Mask R-CNN for crack detection.

Mask R-CNN is a method advantageous for instance segmentation, which means both object detection and semantic segmentation. The Mask R-CNN is chosen because the crack detection task requires the identification of the position box of cracks and distinguishing pixels in the box between the crack and other classifications, such as background, water seepage and rust. The framework of the procedure is shown in Figure 4 where the first step is raw image pre-process, the second step is feature generation which includes feature map and fixes-size feature, and the third step is classification.

The principle of Mask R-CNN is to attach a region proposal network to the network head architecture of Faster-R-CNN to replace in parallel the mask map information for detecting the region of interest (RoI). Firstly, the raw image is preprocessed and treated as input data. Secondly, the data are inputted to a CNN network which has been trained and feature map is obtained. The Resnet-50 and DeepLab system are used in this paper. It is noteworthy that the Mask R-CNN as a universal method is also capable of integrating with other CNN networks, such as ResNeXt and VGG16. Thirdly, the region proposal network is constructed in which fixed-size features are generated. The RoIs are preset for the feature map to achieve the RoI proposals, which then go through classification and bounding-box regression for a preliminary filtering of the objects from the background. Additionally, the RoI Align layer processed the feature map to aligned with pixel and features so that the feature maps are converted to a fixed-size. Fourthly, classification: Bounding-box regression of the RoIs are performed by means of a fully convolutional network.

The Resnet-50, which contains one initial block, 4 stages, average pooling and a fully connected layer, was employed as the backbone. More details are shown in Table 1, where the conv1, conv2_x, conv3_x, conv4_x and conv5_x represent the convolutional layers included in the initial block and stages 1–4, respectively. The conv1 is with 7 × 7 kernel size, 64 channels, and stride 2, followed by max pooling with 3 × 3 kernel size and stride 2. Because there are 16 building blocks in the 4 stages, (3, 4, 6 and 3 blocks in stages 1–4, respectively) and each block contains 3 layers, the 4 stages includes 48 layers in total. The total number of layers comes to 50 putting in the conv1 layer and one fully connected layer. The Resnet-50 is optimized by means of bottleneck design in reducing the number of parameters. The residual block is optimized where two 3 × 3 convolutional layers are replaced with 1 × 1, 3 × 3 and 1 × 1 convolutional layers. The middle 3 × 3 convolutional layer is computed after reducing dimensionality through the first 1 × 1 convolutional layer, and thereafter the dimensionality is recovered by the second 1 × 1 convolutional layer, which is capable to maintain both high accuracy and low computational cost [29].

**Figure 5:**
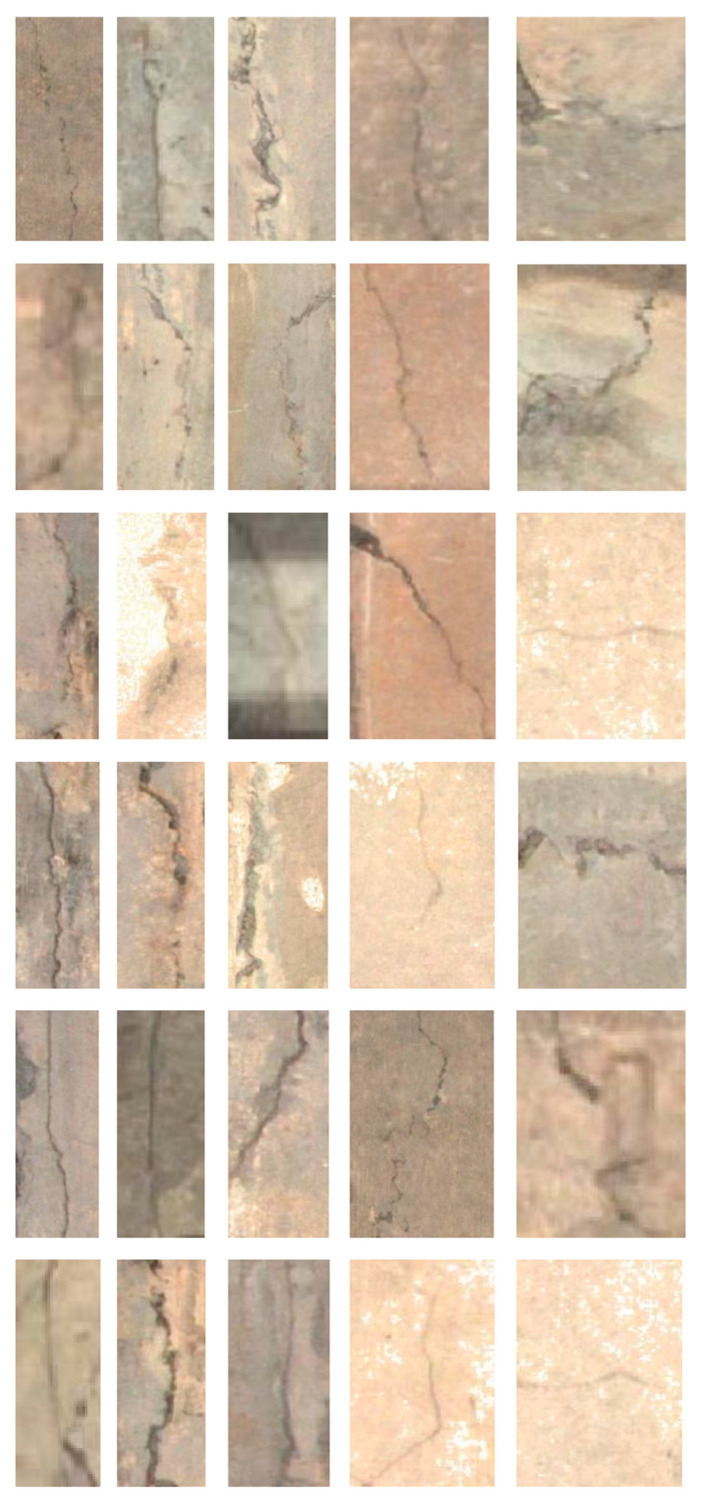
The crack images for the Mask R-CNN algorithm.

**Figure 6:**
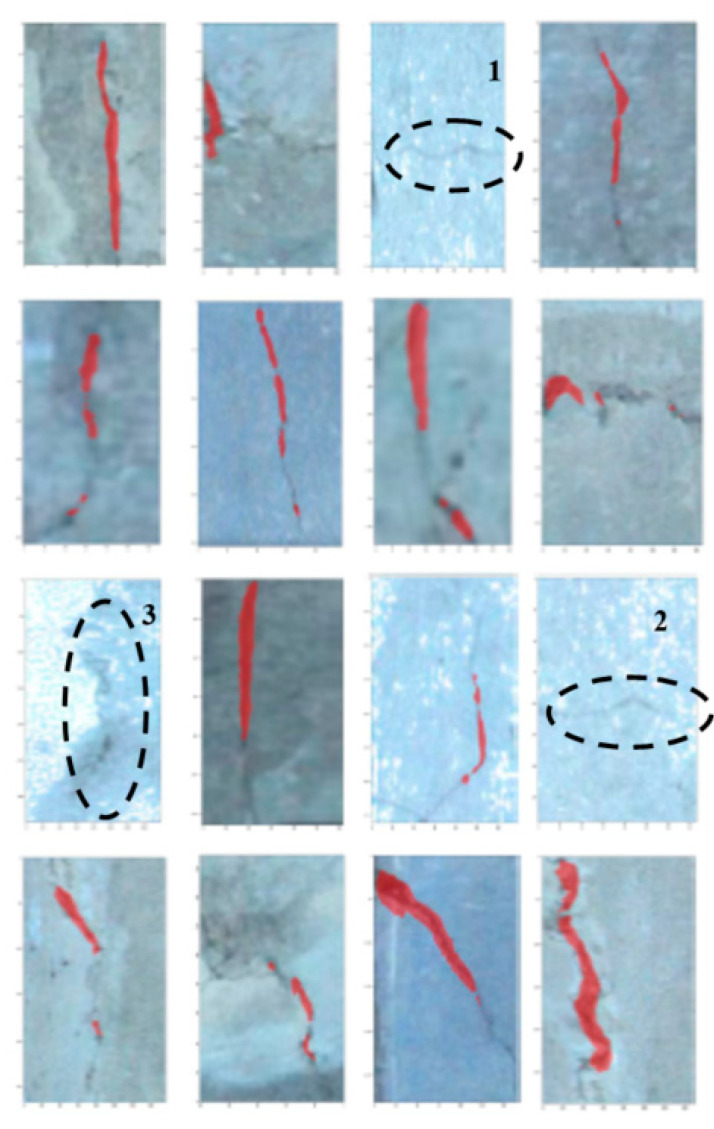
The training dataset of crack.

**Figure 7:**
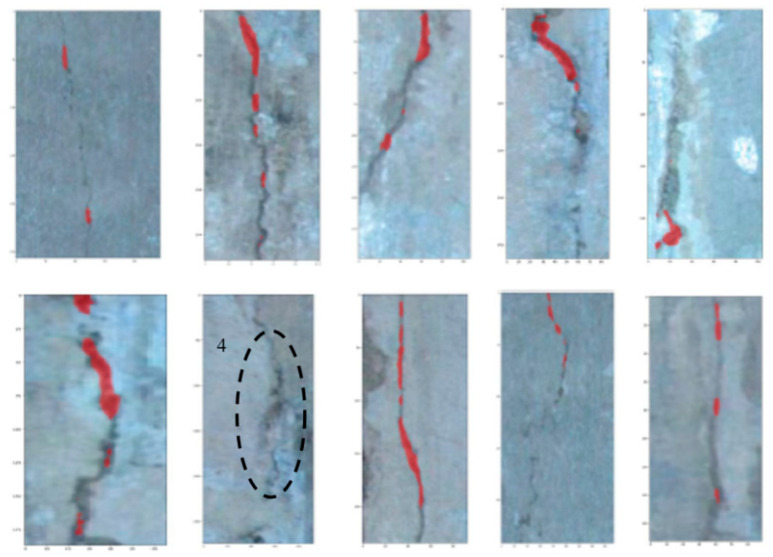
The testing dataset of cracks.

In this investigation, the Mask R-CNN network is applied to preprocessed crack images extracted from the stitched images for crack identification. A total of thirty images are processed and shown in Figure 5, where twenty of them are selected as a training dataset, and the remaining ten images make up the test dataset. The training data correspond to Figure 6 in this section and the testing data correspond to Figure 7 in section four. In order to test the performance of the method, different cracks are chosen in terms of size, direction and shape, and the quantity of total crack images is not large.

The end-to-end training approach was employed [30] and mask R-CNN’s loss function can be expressed as Equation (15).

***L*** = ***L_c_*** + ***L_b_*** + ***L_m_*** (15)

In the Equation (15), ***L_c_*** represents the classification loss of the bounding box, ***L_b_*** represents the regression loss of the bounding box, and ***L_m_*** represents the loss of the mask part.

## 4. Results

In this study, the pretrained model is employed to initialize the parameters of the backbone, while other parameters are initialized through random numbers obeying Gaussian distribution [31], and the mini-batch gradient descent method is adopted to train the model. The training dataset is shown in Figure 6, which can be expanded by randomly increasing the noise and changing the contrast.

In Figure 6 sixteen different cracks, which are collected from the tunnel structures, are selected in the training dataset where the red pixels denote the cracks detected with the Mask R-CNN algorithm. It could be observed in Figure 6 that the vertical cracks are located correctly and the horizontal cracks are neglected; the corresponding images are marked with numbers 1 and 2 in the figure. Furthermore, a higher contrast is beneficial for crack detection, while low contrast images may result in complications in detection, such as the image marked with number 3.

In this analysis, ten images were selected as a testing dataset which can be augmented significantly by randomly changing contrast and adding noise. The testing results are shown in Figure 7.

It is observed in Figure 7 that most cracks are positioned correctly whose pixels are marked in red, however, the image marked with number 4 is not successfully identified. It is indicated that cracks in the fallen area of concrete are more difficult to detect, mainly because the crack features in this area are concealed by other features, so that the feature matrix of the cracks cannot be effectively identified.

**Figure 8:**
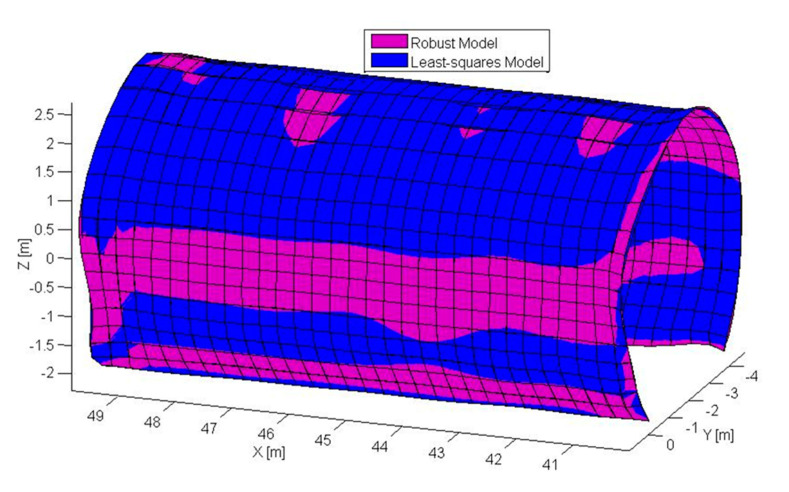
The robust 3D B-spline model for the tunnel.

The point cloud data are processed using a self-developed robust B-spline algorithm. To generate net-wise input data, the data are re-sampled where the data points are chosen through picking out the nearest points to the preset net. Based on the character of the input data, the parameters m, n, p and q of the B-spline model are decided, where the m, n related to the control point number are set to assure that one control point distributes approximately for every one meter, and the degree of basis function p and q are determined equally as three. Furthermore, the 3D model is constructed using the robust B-spline surface method proposed, where the unknown B-spline control point matrix are estimated and the B-spline surface are constructed. The 3D B-spline model generated is presented in Figure 8, which represents a segmentation of 40–50 m in the tunnel.

The 3D model of tunnel structures is constructed and presented in Figure 8, where the purple corresponds to the robust B-spline model which is resistant to the disturbing and noisy points, and the blue one is the least-squares model. The robust B-spline model can automatically eliminate the disturbing points, reserving the tunnel’s geometric features, and efficiently achieved a global parametrization of the tunnel segment.

## 5. Discussion

The quality of point cloud data could be decreased due to various factors, such as the quality of the image collection and the accuracy of the image registration. In order to test the model’s resistance to the decreased quality of point cloud data, the latter is processed through manually filtering the disturbing objects, and the deviation between the filtered point cloud and the B-spline surface points is represented by root mean square error (RMSE). Different segmentations are analyzed and the RMSE of the robust and least-square models are compared, where the results are listed in Table 2.

According to Table 2, the robust model gains significant improvement compared to the least-squares model in terms of RMSE. It means that the robust model has a better performance in the 3D geometric modelling of tunnel structures. It also indicates that a parameterized robust model combined with machine learning can improve the intelligence level of tunnel health monitoring significantly.

For the crack detection, it is demonstrated that higher contrast of images is beneficial for crack detection, while low contrast images may result in complication of the detection. This gives the cue that the hardware of the system should be calibrated so that it collects high-contrast image data in various tunnel environments. Besides, the mixture of features in the images, which could be also understood as a kind of low-contrast case, makes the crack detection more challenging, e.g., the cracks in the fallen area of concrete are more difficult to detect, mainly because the crack features in this area are concealed by other features, so that the feature matrix of the cracks cannot be effectively identified. It is also shown that the different crack features gain unequal detection accuracy, where vertical cracks are mostly located correctly and the horizontal cracks are sometimes neglected. The detection accuracy rate is 85.79%, which is computed by the portions of the correctly detected crack pixels in the total pixels of cracks. However, the CNN method owns much higher accuracy than the traditional image processing method of binarization, whose accuracy is only 19.16% on average. Low-rank and sparse representations of the features will be considered in the Mask R-CNN model in the next step to obtain higher crack detection accuracy. Furthermore, strategies that facilitate feature differentiation may improve detection results, for instance calibrating the measurement system to achieve optimal image contrast.

The advantages of the comprehensive evaluation of geometry deformation and crack damage are: (1) More complete structural information can ensure the reliability of health monitoring; and (2) mutual validation of various models can significantly improve the accuracy of health monitoring, considering the response that the relationship between the geometric model and crack model is liable to achieve, since these two models are based on the same data. Therefore, this proposal may bring in a certain basis for the development of structural tunnel health monitoring and improve the level of intelligence. In the next step, the relation map between the crack image and the 3D model will be constructed, where the cracks can be located and visualized in the 3D model environment. Furthermore, an integration analysis will be conducted combining the crack model and tunnel deformation model, which is the change between two temporal geometrical models. Intelligent decision of principal cracks will be conducted, which are related to substantial damage and can reduce the maintenance cost.

## 6. Conclusions

Tunnel cracks are unavoidable in tunnels of transportation systems. However, traditional methods to monitor cracks are dominated based on manual inspection which is limited by the short inspection period and illumination conditions. In this study, the vision-based method is prominent for the possibility of combination with a robotic system and computer-vision processing. This paper proposed a novel method which combines robust freeform surface modelling and deep learning to efficiently obtain the 3D tunnel structure model and identify the cracks.

(i) A measurement of the tunnel structures is carried out with the vision-based method where ten cameras are installed in the vehicle and collect image data of the inner wall of the tunnel.
(ii) The AI-based crack identification method is investigated in which deep learning based on CNN is employed.
(iii) The 3D freeform surface is generated where the maximum likelihood function is applied to improve the robustness of the modelling.
(iv) The global parameterization of the tunnel from images is computationally efficient, robust against disturbing data and convenient for visualization.
(v) The robust model gains significant improvement compared to the least-squares model in terms of RMSE where the improvements in two segmentations are 429.32% and 425.06%.

In conclusion, a novel method combining robust modelling and AI analysis is proposed which will automatically generate the high-accuracy 3D tunnel structures and extract the position of cracks. This study is dedicated to improving the intelligent monitoring level of tunnel structures where intelligent maintenance of the tunnel structures in the age of increasing tunnel mileage will greatly save labor costs and guaranteed security. In the future investigation, the relation map between the crack image and the 3D model will be constructed, where the cracks can be located and visualized in the 3D model environment.

## Figures and Tables

**Table 1:** The layers of Resnet-50 [29].

Layer Name	Details	Output
conv1	7 × 7, 64, stride 2	112 × 112
conv2_x	3 × 3 max pooling, stride 2	56 × 56
	$\left[\begin{array}{c} 1\times 1,64\\ 3\times 3,64\\ 1\times 1,256 \end{array}\right]\times 3$	
conv3_x	$\left[\begin{array}{c} 1\times 1,128\\ 3\times 3,128\\ 1\times 1,512 \end{array}\right]\times 4$	28 × 28
conv4_x	$\left[\begin{array}{c} 1\times 1,256\\ 3\times 3,256\\ 1\times 1,1024 \end{array}\right]\times 6$	14 × 14
conv5_x	$\left[\begin{array}{c} 1\times 1,512\\ 3\times 3,512\\ 1\times 1,2048 \end{array}\right]\times 3$	7 × 7
	Average pooling + Fully connected layer	1 × 1

**Table 2:** The comparison of robust and least-squares models.

Model/Point Cloud Datasets	Segmentation 1	Segmentation 2
Least-squares Model	18.95	23.26
Robust Model	3.58	4.43
RMSE improvement	429.32%	425.06%
